# Retraction: Activation of TGF-β1 Promoter by Hepatitis C Virus-Induced AP-1 and Sp1: Role of TGF-β1 in Hepatic Stellate Cell Activation and Invasion

**DOI:** 10.1371/journal.pone.0216025

**Published:** 2019-04-30

**Authors:** The PLOS ONE Editors

Following publication of this work [1], concerns were raised about the following:

- Fig 2E Actin panel appears similar to lanes 2–4 of the GAPDH panel in Fig 7D. These panels also appear similar to images in Fig 2A (Actin), Fig 2B (Actin), Fig 4A (NS5A), 4B (NS5A), 7D (Actin), and 9A (Actin panel) of a 2003 article published in the *Journal of Biological Chemistry* [2] by the corresponding author of [1]. The 2003 publication has been withdrawn [3], and the withdrawal notice for that article notes similarity of the image in question to figures in several other articles.
- Fig 7B Albumin panel lanes 2,3 flipped horizontally appear similar to Fig 1A ASC panel lanes 3,4 in a 2016 article published in the *Journal of Biological Chemistry* [4] by two of the authors of [1]. The 2016 article has been withdrawn [5], and the withdrawal notice indicates similarity between this image and figures published in several other articles, including two published in 2010 and 2012 [6, 7].

While the authors stand by the validity of the experimental data and the conclusions reached in the study, they were unable to provide original images or underlying data for Figs 2E, 7D and 7E.

In light of the image concerns and the unavailability of the underlying data, the *PLOS ONE* Editors retract this article.

GW did not agree with retraction. LDP and SM did not respond.

Owing to the concerns about similarities with previously published content [2–7] that is not licensed for reproduction and distribution under the terms of the Creative Commons Attribution License, Fig 2E Actin panel, Fig 7B Albumin panel, and Fig 7D GAPDH panel have been removed from the retracted article [1].
